# DeepLocate: Smartphone Based Indoor Localization with a Deep Neural Network Ensemble Classifier

**DOI:** 10.3390/s20010133

**Published:** 2019-12-24

**Authors:** Imran Ashraf, Soojung Hur, Sangjoon Park, Yongwan Park

**Affiliations:** 1 Department of Information & Communication Engineering, Yeungnam University, Gyeongbuk, Gyeongsan-si 38541, Korea; ashrafimran@live.com (I.A.); sjheo@ynu.ac.kr (S.H.); 2 Electronics and Telecommunications Research Institute, Daejeon 34129, Korea; sangjoon@etri.re.kr

**Keywords:** indoor localization, magnetic field, ensemble classifier, smartphone sensors, deep learning, feature extraction, neural networks

## Abstract

A quickly growing location-based services area has led to increased demand for indoor positioning and localization. Undoubtedly, Wi-Fi fingerprint-based localization is one of the promising indoor localization techniques, yet the variation of received signal strength is a major problem for accurate localization. Magnetic field-based localization has emerged as a new player and proved a potential indoor localization technology. However, one of its major limitations is degradation in localization accuracy when various smartphones are used. The localization performance is different from various smartphones even with the same localization technique. This research leverages the use of a deep neural network-based ensemble classifier to perform indoor localization with heterogeneous devices. The chief aim is to devise an approach that can achieve a similar localization accuracy using various smartphones. Features extracted from magnetic data of Galaxy S8 are fed into neural networks (NNs) for training. The experiments are performed with Galaxy S8, LG G6, LG G7, and Galaxy A8 smartphones to investigate the impact of device dependence on localization accuracy. Results demonstrate that NNs can play a significant role in mitigating the impact of device heterogeneity and increasing indoor localization accuracy. The proposed approach is able to achieve a localization accuracy of 2.64 m at 50% on four different devices. The mean error is 2.23 m, 2.52 m, 2.59 m, and 2.78 m for Galaxy S8, LG G6, LG G7, and Galaxy A8, respectively. Experiments on a publicly available magnetic dataset of Sony Xperia M2 using the proposed approach show a mean error of 2.84 m with a standard deviation of 2.24 m, while the error at 50% is 2.33 m. Furthermore, the impact of devices on various attitudes on the localization accuracy is investigated.

## 1. Introduction

Indoor localization has become one of the potential research areas in the last decade. The proposal of RADAR [1] pioneered the indoor positioning with utilizing the radio signals. The emergence of smartphones in 21st century paved the way for the flourishing of localization. The inception and penetration of location-based services (LBS) further accelerated the research in the field of indoor localization. Today, LBS are offered to a large number of users, both indoors and outdoors. The global positioning system (GPS) can achieve localization accuracy ranging from 17 m to better than a few meters [2]. However, this accuracy depends upon many factors including the number and geometry of collected observations, mode, and type of observation, measurement model, level of used biases, design of GPS receiver, and receiving land structure like obstacles or no obstacles [2,3]. The GPS is used for outdoor positioning, yet its sensitivity to occlusions including ceilings and walls makes it inappropriate and inefficient for indoor localization. Although GPS can be used for indoor localization when the user is close to wide windows and the receiver can get signals, the provided location may have a higher error which can in certain scenarios be larger than the indoor localization area itself. The lower signal-to-noise ratio and multipath phenomena result in a less reliable position [4]. This led researchers to investigate alternative technologies that could potentially overcome such limitations and work efficiently for indoor environments.

A large body of work has been presented on such technologies including ultra-wideband (UWB) [5], radio frequency identification (RFID) [6], Wi-Fi [7], and vision [8], etc. Such technologies are however limited by their dependence on additional hardware (with the exception of vision), which needs to be installed in the area intended for localization. Additionally, the wide applicability is restricted by the shortcomings and software and hardware limitations of these techniques. For example, RFID is based on short-range communication and works only in a small area where RFID tags have been installed. The UWB based indoor localization systems provide precise position information but are expensive. Additionally, in the complex and occluded environments, more nodes are required to achieve higher accuracy which further increases the cost [9]. Vision-based indoor localization although it does not need additional infrastructure, but requires a significant amount of computational resources to perform the image matching. The modern graphics processing unit (GPU) can do the image matching in a reduced amount of time; however, vision-based systems’ performance is degraded in case of low lighting conditions and poor image quality which can happen with the phone holding orientation of the user.

The proliferation and wide usage of modern smartphones present a potential solution to many of the above-mentioned limitations. Today, modern smartphones are equipped with a variety of sensors that can be leveraged to perform indoor localization. Smartphone sensors including Wi-Fi, Bluetooth, and camera resulted in the development of many localization techniques. Wi-Fi and Bluetooth based localization systems are limited by inherent limitations of wireless communication e.g., the propagation losses and environmental changes cause substantial changes in received signal strength (RSS) [1,10,11]. The problems of multipath shadowing, signals fading, and impact of other dynamic factors including human mobility on signal fluctuation may lead to very high localization error. In the same fashion, the impact of human body loss causes signals absorption and the change in the RSS leads to higher localization error [12]. Additionally, the RSS has been found to be dependent on hardware and an antenna design which may be an inherent limitation of Wi-Fi positioning accuracy [13]. The sensors embedded in the smartphone are utilized in a variety of practical tasks. The authors present an object classification framework in [14] using the hyperspectral camera. Similarly, wearable sensors are utilized as well in many practical applications. A triaxial accelerometer-based human motion detection system is proposed in various research works [15,16]. The features extracted from the data are used in machine learning-based models for that purpose. Feature extraction for such applications is very important. Thus, we can find various works which aim at finding various features for these tasks. Research [17] works on feature extraction on the unmanned aerial vehicles (UAV). Algebraic representation of Spatio-temporal real-world objects is presented in [18]. Local descriptors are used to track a person in two different cameras with support vector machines (SVM) [19]. In the same way, similar human interactions are recognized with a supervised framework in [20]. Motion estimation is another important task in today’s real-world applications and we can find a large body of work about human activity detection [21], motion estimation, and real-time motion detection through multiple cameras [22,23]. Various sensors have been employed to achieve such tasks. For example, the authors in [24] present a geometric-constrained multi-view image matching method that aims at the efficient and reliable processing of multiple remote sensing images. Machine learning [25], as well as deep learning frameworks [26], have also been utilized for human and human interaction detection.

The geomagnetic field-based localization has emerged as a new paradigm during the last few years [27,28,29]. Today, a large body of works [30,31,32,33] can be found which utilizes the earth’s magnetic field data for indoor localization. The geomagnetic field (referred to as the magnetic field for convenience) is the natural phenomenon that is caused by the flow of convection current in the outer layer of the earth. The magnetic field is a vector field and possesses a direction and magnitude. It requires three parameters to represent the magnetic field at a point. The north, east, and downward components are represented *x*, *y*, and *z*. A common way to represent the magnetic field is with the total intensity *F*, the inclination *I*, and the declination *D*. However, the most widely used representation is through magnetic *x*, *y*, *z*, and *F*. Another way of showing the magnetic field is through the horizontal component *H*, the vertical component *z*, and the declination *D* [34]. The total magnetic strength on the earth’s surface varies from 25 micro Tesla to 65 micro Tesla [35]. The magnetic strength and its direction do not change over a small restricted area, yet man-made construction obstructs the magnetic field and alters it to cause magnetic disturbances. Such magnetic disturbances are called anomalies and observed to exhibit unique behavior. These magnetic anomalies have been studied and used as a fingerprint in many research works [30,36]. However, the techniques which utilize magnetic field fingerprints have two major limitations. First, owing to the use of various magnetometers in heterogeneous smartphones, the magnetic strength is different even for the same location [27]. This limits the wide applicability of magnetic field based localization systems as making a common fingerprint for various smartphones is not possible. As a result, the localization error is different even when a single localization approach is adopted for various smartphones. Second, multiple distant locations may have a very similar magnetic signature due to the indoor environment. It is highly probable, especially when the localization space is large. On account of the above-mentioned shortcomings, the fingerprinting technique is not suitable for indoor positioning where magnetic data are used. This study aims to leverage deep neural networks (NN) to address these issues.

Deep learning has recently been utilized to solve many problems and indoor localization is no exception. Deep NN and convolution neural networks (CNN) have been used for indoor scene recognition, object detection, and localization, etc. However, a single NN may perform worse in case of noisy data. This is why this study proposes the use of an ensemble that is based on multiple NN that are trained separately. The prediction from each of these classifiers is then employed to find the final location of the user. Deep learning is a data-intensive technique and requires a large amount of data for training. For this purpose, thousands of magnetic samples have been collected. The key contributions of this research can be summarized as:

- A deep neural network (NN) based approach is presented which performs the indoor localization based on the features extracted from the magnetic data.
- A soft voting criteria is defined to ensemble the prediction of multiple NNs. All NNs are trained with the same magnetic data to predict the user’s current location.
- The proposed approach is tested with heterogeneous devices including Galaxy S8, LG G6, LG G7, and Galaxy A8 to evaluate the localization accuracy. The results are compared with support vector machines (SVM) and another magnetic localization approach.
- Besides our own collected dataset, the proposed approach is tested on a publicly available magnetic dataset where the data have been collected with a Sony Xperia M2 smartphone.
- The impact of device varying attitude has also been investigated where the device attitude is changed from ’navigation’ to ’call listening’, and ’front pocket’ mode to analyze the localization performance of the proposed approach.

The rest of the paper is organized in the following manner. Section 2 presents an overview of a few studies related to this research. The current challenges of magnetic field based positioning are discussed in Section 3. Section 4 describes the proposed approach while Section 5 details the experiment setup and analyzes the results. Finally, conclusions are given in Section 6.

## 2. Related Work

The application of magnetic field data for indoor localization has been investigated by many research works. Such research includes the analysis of properties of magnetic field data that can be used for localization, as well as the impact of various devices usage, and the attitude of these devices [28,30,36]. Research works using the magnetic field can broadly be categorized into three groups: using magnetic field data alone, hybrid approaches that combine magnetic field with Wi-Fi, pedestrian dead reckoning (PDR), vision, etc., and approaches that utilize machine/deep learning. A few works related to each category are discussed here.

Authors in [37] investigated the use of a smartphone magnetometer to perform indoor localization. The investigation is aimed at studying the localization performance of magnetic data alone. The localization error is low if more elements of the magnetic field are used. However, the error may rise up to 20 m when the localization area is large and complicated by structure. The proposed technique is based on the fingerprint database of magnetic field data, which is laborious and time-consuming. Authors in [31] used the crowdsourcing approach to build the fingerprint and minimize the labor and cost involved in fingerprinting. They employed a revised Monte Carlo technique to locate a pedestrian indoor. The proposed approach is able to converge to a 5 m area by using 30 s data. The research suggests that localization error using magnetic field data alone is higher and other assistive technologies could help to lower the error. Therefore, many research works focusing on the use of magnetic field data with other localization techniques can be found.

For example, an indoor localization system is presented in [38] that combines Wi-Fi signals with the magnetic field data to build the fingerprint database. Initially, Wi-Fi access points (AP) are used to calculate an approximate location. This position is later used to restrict the search space that helps reduce the localization error to 4.5 m. The use of magnetic field data alone with the proposed technique results in an error of up to 16.6 m. Similarly, authors in [39] present an approach that is based on the fusion of magnetic field data with PDR. An artificial neural network is used to identify the user walking and stationary modes. The user movement is tracked at regular intervals and its relevant position is utilized to refine the magnetic position. The reported accuracy is 2–3 m at 50% with two different smartphones. Furthermore, an approach is proposed in [40] which works with PDR and magnetic data to locate a user in the indoor environment. An approach similar to particle filter has been adopted which takes into account the PDR and magnetic position of the user and predicts the final location of the user. Experiment results show under 2 m accuracy with two different devices.

Research proves that the fusion of more than one localization technology can significantly improve the localization accuracy. For instance, authors in [41] present a system called WAIPO. The system is based on the fusion of Wi-Fi and magnetic fingerprints, image matching, and people’s co-occurrence. Initially, the position is estimated using Wi-Fi fingerprints which can further be refined with image matching and Bluetooth beacons. The final position is then calculated with the magnetic data from the user smartphone. The reported accuracy of WAIPO is under 2 m at 98 percent.

Various machine learning models have been proposed as well for human detection in an indoor envrionment. Such models work on various features extracted from the sensor’s data and perform human and object detection. For example, research works [42,43] focus on human interaction recognition with the help of artificial neural networks. The genetic algorithm is applied to identify prominent objects under varying environmental settings. Likewise, authors in [44] make use of graph kernel-based SVM and bag-of-words to perform abnormal activity detection. Authors investigate the use of K-means++ and support vector data description in [45] to cluster the data for regions of interests. Additionally, the use of pyroelectric infrared sensors is reported to perform abnormal activity detection in [46]. The similarity between normal training samples is measured using Kullback–Leibler divergence, and one-class SVMs are used to perform the activity detection. Similarly, the use of depth information using the depth sensors has been proven to increase human activity recognition and tracking in smart houses [47,48].

Recently, the use of deep learning has been reported to perform localization with smartphone sensors. Authors in [49] propose a system that makes use of a variety of smartphone sensors to localize a pedestrian. The research uses a smartphone camera, motion sensors, compass, magnetometer, and Wi-Fi to do the localization. A CNN has been designed that can identify the indoor scene. The recognized scene is later used to narrow down the search space in the magnetic database. The reported localization error is 1.32 at 95%. In the same fashion, the research [50] proposes a multi-story localization approach based on smartphone sensors. The smartphone camera is utilized along with the magnetometer. Instead of magnetic intensity, the magnetic patterns are used to build the database. Smartphone camera pictures have been used for indoor scene recognition. The CNN model helps to identify a specific floor. It also increases the localization accuracy by narrowing down the magnetic database search space. The reported localization error is 1.04 m at 50 percent. CNN has been utilized with the magnetic data as well to perform the localization. For example, authors in [51] present a magnetic field-based indoor localization method that utilizes CNN with smartwatch. The magnetic data along with smartwatch orientation data are used for training. Experiment results show promising results. The NN has been utilized in Wi-Fi based localization as well. Authors in [52] propose a stacked denoising auto-encoder based feature extraction to extract Wi-Fi fingerprints to perform localization. The proposed approach tackles the problem of RSS fluctuation in dynamic environments and improves the localization accuracy.

The above-mentioned research works are limited by two factors in essence. The first problem lies in the use of Wi-Fi signals, which, as already discussed, are vulnerable to propagation loss. Furthermore, the RSS value is subject to dynamic factors including the presence of obstacles, shadowing, and human mobility. Secondly, the impact of device heterogeneity is not studied very well. Very few studies consider device heterogeneity, yet they, in turn, use longer data samples. For example, the authors in [39] consider 14 s data while the authors in [40] employ 8 s data to calculate the final location of the user. Additionally, the use of a smartphone camera consumes the battery very fast and is not an efficient solution. Similarly, the camera has its own inherent limitations including image quality under poor light conditions and dark environments. It is noteworthy to point out that deep learning has been utilized on smartphone camera images alone. This study aims to use deep neural networks on the magnetic field data to perform indoor localization.

## 3. Current Challenges in Magnetic Field Based Localization

### 3.1. Indoor Infrastructure and Time Variance

Although the magnetic field has proven to be time-invariant, yet it is highly affected by the indoor infrastructural change, especially those involving ferromagnetic material like iron, steel, etc. Similarly, the addition of steel doors and cupboards cause changes in the magnetic field strength [27]. Additionally, the placement of refrigerators and vending machines in corridors also tend to have a considerable impact on the magnetic field.

### 3.2. Heterogeneity of Smartphones

**Figure 1:**
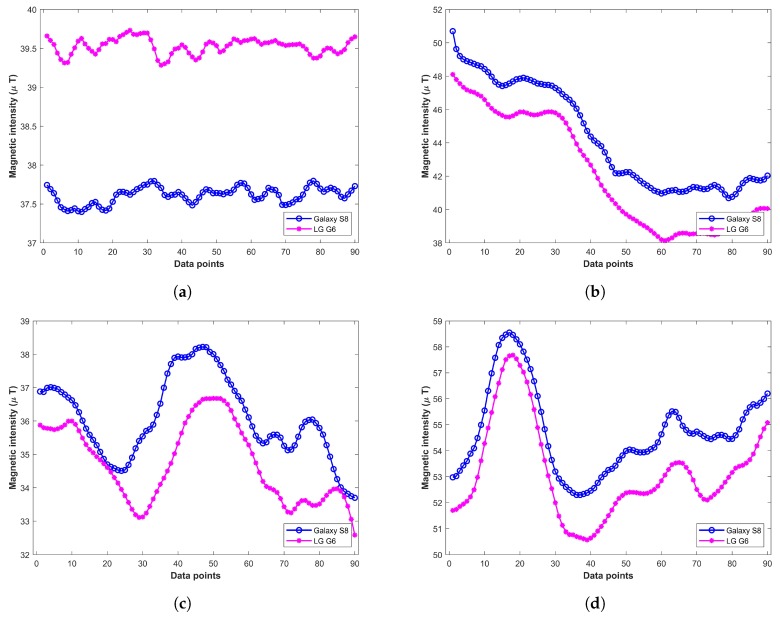
Magnetic readings from various smartphones at different indoor locations, (**a**) Location 1; (**b**) Location 2; (**c**) Location 3; (**d**) Location 4.

The magnetic field readings differ with different devices even for the same magnetic field [27]. Hence, it is a big challenge to make a localization system that can work with the heterogeneous device in the same fashion, as we have diverse categories of devices today. This is shown in Figure 1 where the magnetic readings from Samsung Galaxy S8, and LG G6 are plotted. Each subplot shows the magnetic readings from two devices at a separate location. The magnetic readings are collected standing at the same place with the smartphone held in the hand in front of the body.

### 3.3. Various Device Attitudes

**Figure 2:**
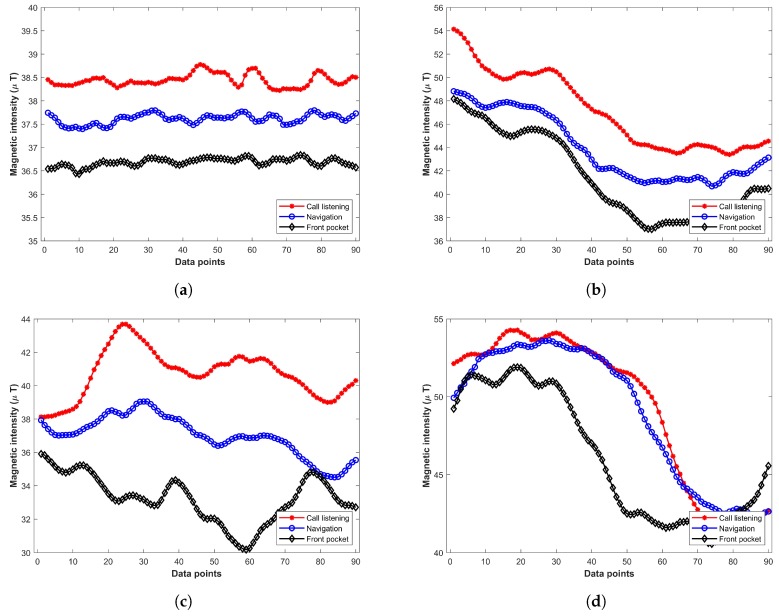
Magnetic readings with various attitudes of device at different places, (**a**) Location 1; (**b**) Location 2; (**c**) Location 3; (**d**) Location 4.

The various attitudes of even the same devices make a significant change in the magnetic readings. For example, Figure 2 shows the magnetic readings taken from Galaxy S8 at various locations with different orientations. Each subgraph shows the magnetic readings at the same location. The readings are collected for three attitudes including ‘navigation’, ‘call listening’, and  ‘front pocket’. It shows that the total magnetic intensity for various orientations is different. Experiments show that the change in total magnetic intensity is minimal compared to that of *x* and *y* components. Thus, for a magnetic field-based positioning system that intends to utilize magnetic *x*, *y*, and *z* components, the common assumption is to fix the attitude of the device [27,53,54]. Traditionally, the phone is held in the hand in front of the body while the user walks.

### 3.4. Low Strength of the Magnetic Field

The magnetic field strength is commonly very weak and measured in μT. Thus, it is possible to observe the same magnetic field strength at a number of locations in the indoor environment. Although the magnetic field is directional and 3D magnetic signals in *x*, *y*, and *z* directions can be utilized for positioning, in practice, it is difficult to implement, as it requires the tracking of device attitude during the positioning process.

## 4. Materials and Methods

This section provides the details of the proposed approach. The proposed approach is based on the use of deep learning to train NNs for localization. The first task is to find suitable features that are fed into the NNs.

### 4.1. Features Selection

The major limitation of using the magnetic field is the device dependence. The intensity of collected magnetic data may be different depending on the sensitivity of the installed magnetometer in various smartphones.

**Figure 3:**
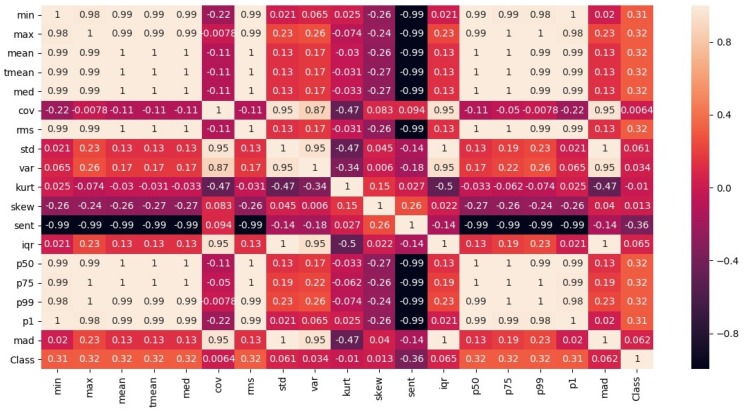
Correlation of selected features to predict a specific class. ’Class’ weight in rows shows the importance of features.

Another shortcoming of magnetic data is its low dimensionality. The magnetic *x*, *y*, and *z* are traditionally used to build the fingerprint. These values may be very similar in multiple locations, especially in a large space. Thus, contrary to using the magnetic field data, this study aims to work with the important features of this data. Initially, a total of 18 features, as shown in Table 1, are shortlisted. Then, a feature analysis is performed and the correlation of each feature to the prediction is evaluated, upon which features including ‘coefficient of variance’, ’kurtosis’, ’Shannon’s entropy’, and ’skewness’ are dropped due to their little correlation to the classification label. The correlation of the features is shown in Figure 3.

### 4.2. Proposed Approach

**Figure 4:**
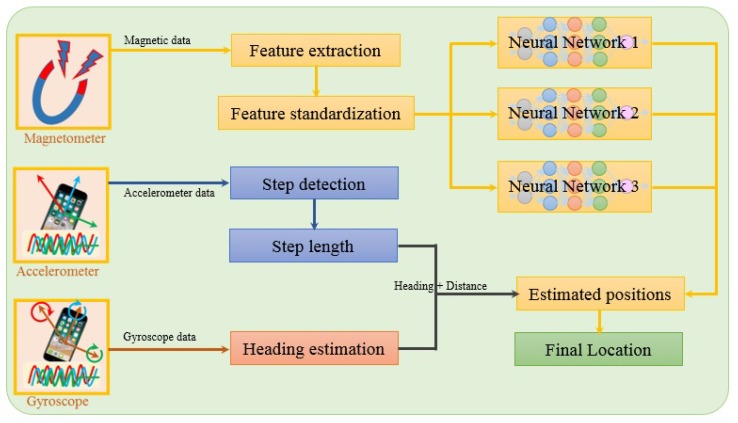
Architecture of the proposed approach.

**Figure 5:**
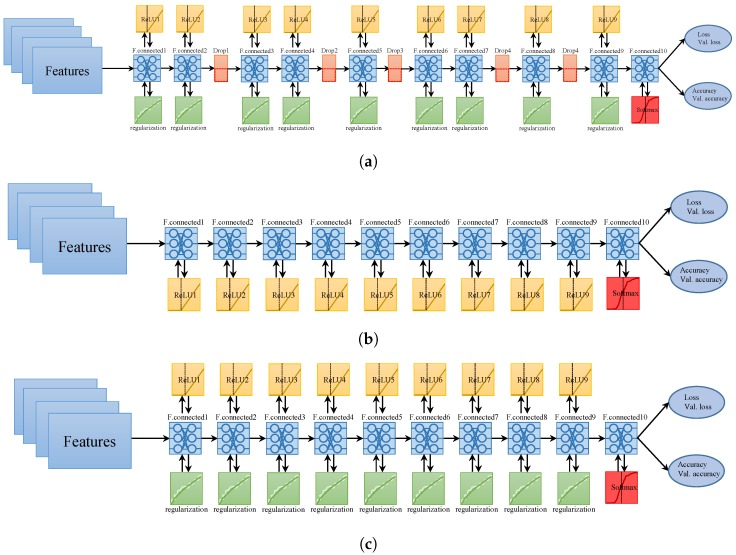
Structure of neural networks (NN), (**a**) $NN_{1}$; (**b**) $NN_{2}$; (**c**) $NN_{3}$.

The architecture of the proposed approach is shown in Figure 4. The features extracted from the magnetic data are fed into neural networks. In addition, two other sensors including the accelerometer and gyroscope have been utilized to approximate the user’s relative motion and direction. Three different NNs make use of magnetic features to predict the user’s position. The purpose of using three different NNs is to leverage the predictions such that the prediction accuracy can be maximized. Each NN has a different architecture in terms of the number of hidden layers, the sequence of layers, and activation functions. The structure of each NN is shown in Figure 5. Each NN is comprised of different numbers of hidden layers, as well as the placement of dropout and regularization layers. The features extracted from the magnetic data are used to train the neural networks.

In the same fashion, during the positioning phase, first of all, the features from the user collected data are extracted and fed into each NN to get the prediction. The user data are collected for three consecutive frames at a sampling rate of 10 Hz/s, where each frame is comprised of 2 s. Pre-processing plays an important role in the prediction process. The noise in the training data degrades the performance of the classification models. The data from smartphone sensors contain noise, so pre-processing is performed to clean this noise. For this purpose, a low pass filter has been used on the sensor’s data before the feature extraction. The positioning process follows the steps given in Algorithm 1. Here, each step is described in detail.

Step 1 (line 1): The first step is to get the predictions from three NNs. For this, instead of a single prediction from each NN, top *k* classes with highest probability are selected where value of *k* is 10. The value of *k* is based on the empirical findings. The predictions are collected for $T_{1}$ and denoted as $P_{NN1_{T_{1}}}$, $P_{NN2_{T_{1}}}$, and $P_{NN3_{T_{1}}}$ for $NN_{1}$, $NN_{2}$, and $NN_{3}$, respectively.

**Algorithm 1** Find user location
1:get predictions $\left(P_{NN1_{T_{1}}},P_{NN2_{T_{1}}},P_{NN3_{T_{1}}}\right)$ from NN.  2:
**for** $i⟵\;1\;to\;length(P_{NN1_{T1}})$ **do**
3: **for** $j⟵\;1\;to\;length(P_{NN2_{T1}})$ **do**
4:  $d⟵\;calDist(P_{NN1_{T1}}\left[i\right],P_{NN2_{T1}}\left[j\right],P_{NN3_{T1}}\left[j\right]$  5: **end for**6: $L_{c}⟵findLocCandidates\left(d,P_{NN1_{T_{1}}},P_{NN2_{T_{1}}},P_{NN3_{T_{1}}}\right)$  7: $L_{c}⟵removeDuplicates\left(L_{c}\right)$ 8:**end for**  9:
**for** T⟵2to3 **do**
10: $L_{c}⟵updateLocCandidates\left(L_{c},Sl_{T},\psi_{T}\right);$   11: $L_{c}⟵refineLocCandidates\left(L_{c},P_{NN1_{T}},P_{NN2_{T}},P_{NN3_{T}}\right);$   12:**end for** 13:
$L_{p}⟵calCenteroid\left(L_{c}\right);$



Step 2 (lines 2–8): Once top *k* predictions have been taken from each NN, a voting mechanism is needed to combine the predictions. For this purpose, the Euclidean distance *d* between the predictions is calculated and a soft voting scheme is followed. A threshold α is set to select the common predictions from three NN. The value of α is 2 m and location candidates $L_{c}$ are selected with the following criteria:

(1) $$L_{c}=\left\{\begin{array}{cc} if\;d<=\alpha & \mathrm{select}\;\mathrm{predictions}\;\mathrm{from}\;P_{NN1_{T_{1}}},P_{NN2_{T_{1}}},P_{NN3_{T_{1}}},\\ otherwise, & \mathrm{drop}\;\mathrm{prediction}. \end{array}\right.$$

Equation (1) states that, if the distance between one prediction by $NN_{1}$ and any of the predictions by $NN_{2}$, and $NN_{3}$ is less than or equal to 2 m, then all the predictions from $NN_{1}$, $NN_{2}$, and $NN_{3}$ are added in $L_{c}$. Since three NNs may have the same predictions, duplicate predictions from $L_{c}$ are removed.

**Figure 6:**
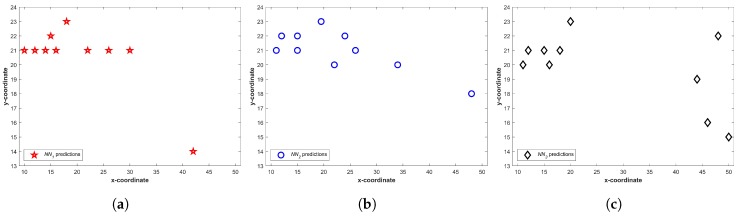
Predictions from three neural networks, (**a**) $NN_{1}$; (**b**) $NN_{2}$; (**c**) $NN_{3}$.

**Figure 7:**
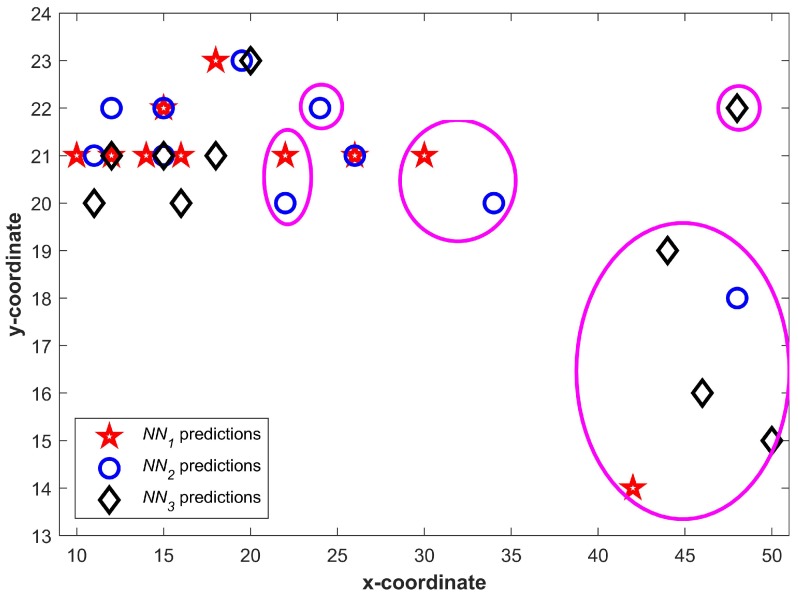
All predictions from neural networks drawn together.

**Figure 8:**
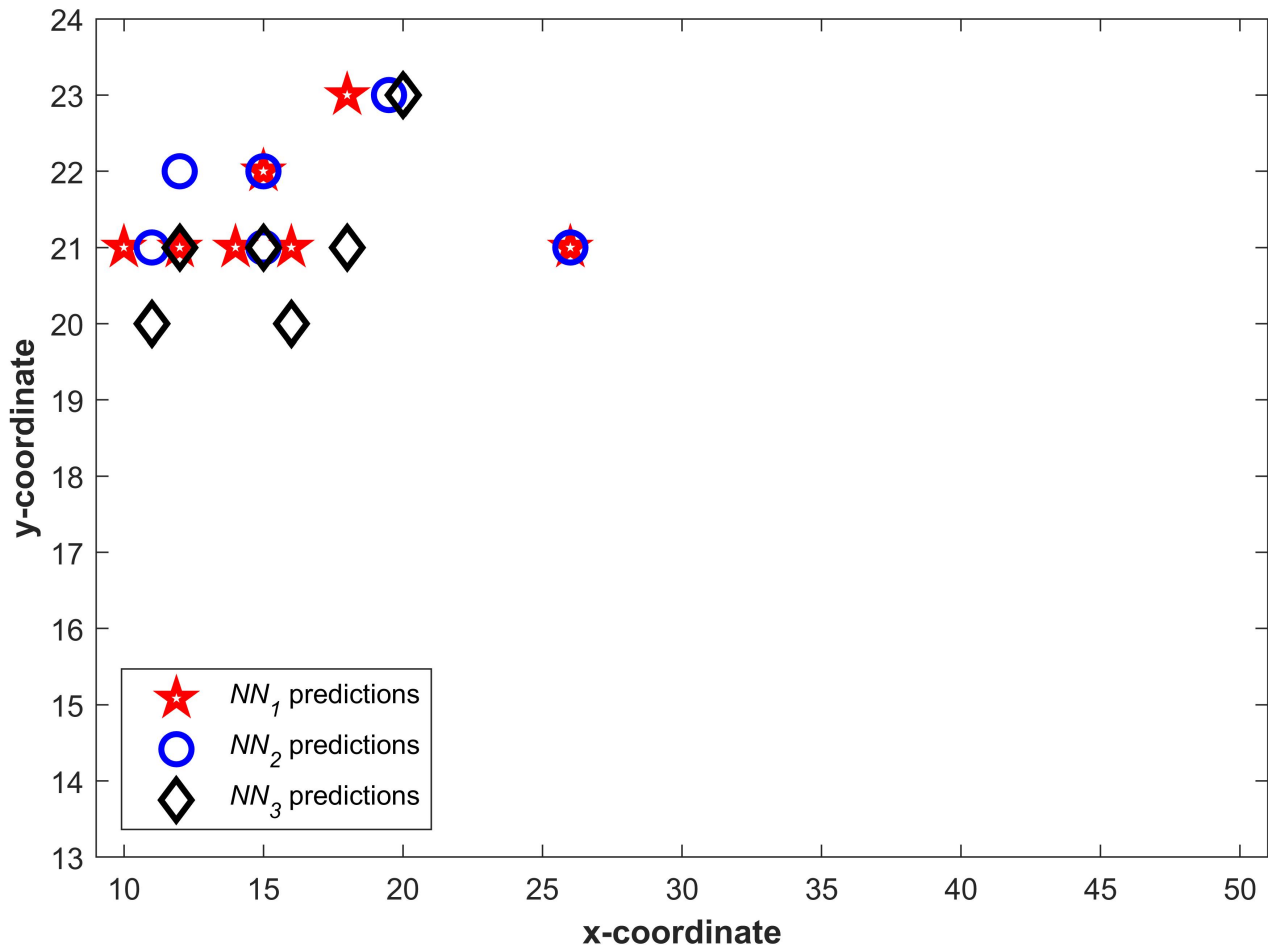
Selected predictions from neural networks after using Equation (1).

Figure 6 shows the predictions from three NNs for $T_{1}$. We can see that a higher number of predictions fall in a small area and few of them may even overlap. It becomes more vivid when predictions are drawn together. Figure 7 shows all predictions drawn together. The predictions which are closer in spatial dimension can easily be seen. Once Equation (1) is applied, the predictions not shared by three NNs can easily be identified. The circled predictions in Figure 7 represent the predictions which are only made by either of NNs and not shared by other NNs (They do not fulfill the criteria set in Equation (1). Once the predictions which do not fulfill the criteria given in Equation (1) are removed, the selected $L_{c}$ can be seen in Figure 8.

Step 3 (lines 9–12): Now, $L_{c}$ are updated for $T_{2}$, and $T_{3}$ with the help of user estimated step length and heading estimation. User approximate relative position is calculated to this end. For this purpose, user step length estimation and heading are required. Step detection is performed using the method proposed in [39], while step detection is done with Weinberg model [55]:

(2) $$Sl=k\sqrt[{4}]{a_{max}-a_{min}},$$

where $a_{max}$ and $a_{min}$ show the maximum and minimum acceleration during a time period. Once step length estimation is done, the position $x_{T}$, $y_{T}$ can be calculated using $Sl_{T}$ and heading estimation $\psi_{T}$ as follows:

(3) $$\begin{array}{c} x_{T}=x_{T-1}+Sl_{T-1}\times cos\left(\psi_{T-1}\right), \end{array}$$

(4) $$\begin{array}{c} y_{T}=y_{T-1}+Sl_{T-1}\times sin\left(\psi_{T-1}\right), \end{array}$$

where $x_{T}$ and $y_{T}$ give the approximate relative position and show only how much the user has traveled in a particular direction during time *T*. The $L_{c}$ can be updated using the approximated position of the user.

After that, $L_{c}$ are refined with the predictions from NN for $T_{2}$. The refining criterion is the same as given in Equation (1); however, *d* is now calculated using $L_{c}$ and NN predictions. As mentioned before, each *T* is comprised of the data of 2 s, and, at a moderate speed, the user can travel up to a 2 m distance during the considered time window. Thus, if the new predictions are within the range of 2 m from the locations given in $L_{c}$, they are selected; otherwise, they are dropped. Since the distance data may contain an error due to noise, a compensating factor ϵ is introduced in the α threshold and its value is 0.18 m. The value of ϵ is based on the error found during experiments and represents the average error in pedestrian dead reckoning (PDR) estimation over 2 s. Now, the value of α is α+ϵ, and $L_{c}$ is defined as follows:

(5) $$L_{c}=\left\{\begin{array}{cc} if\;d<\alpha , & \mathrm{select}\;\mathrm{predictions},\\ otherwise, & \mathrm{drop}\;\mathrm{prediction}. \end{array}\right.$$

The same process is repeated for $T_{3}$ where new $L_{c}$ are updated with PDR data, and new predictions from NNs are refined with respect to $L_{c}$.

Step 4 (line 13): After this step, location candidates $L_{c}$ converge to a small area. Now, their centroid is calculated which gives the user’s current predicted location $L_{p}$.

## 5. Results and Discussion

This section describes the experiment set up used and various scenarios followed during the experimentation. It also analyzes the results of the experiments.

### 5.1. Experiment Set Up

**Figure 9:**
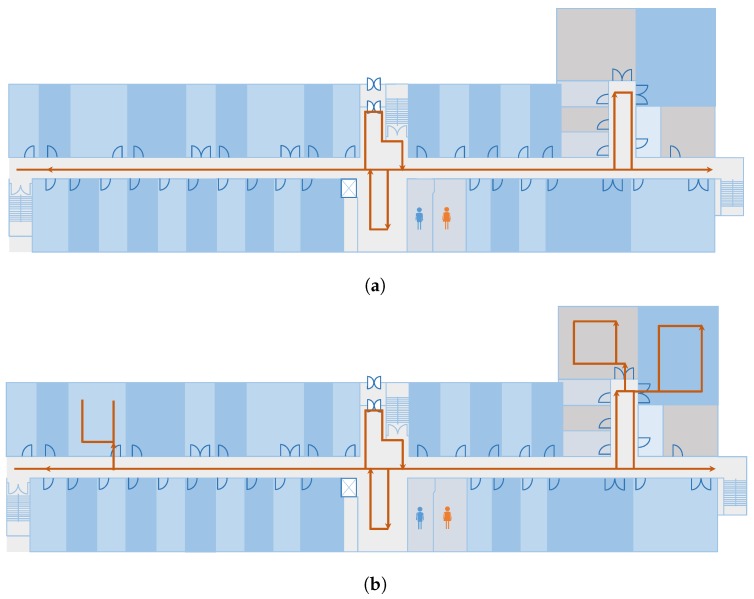
The path used for the experiments for, (**a**) Scenario 1 and (**b**) Scenario 2.

The experiments are conducted in the Information Technology (IT) Engineering building at Yeungnam University, Korea. The dimensions of the building where the localization is performed are 92 × 36 m^2^. Two scenarios are tested using the proposed approach as shown in Figure 9a,b. The first scenario includes the testing in the corridor space only while scenario 2 includes three laboratories as well. The laboratories are added on account of their availability, while other rooms and offices are not open for experiments. The same path is followed in the forward and backward direction. The magnetic data collection is carried out with a Samsung Galaxy S8 (SM-G950N) device (Samsung Seoul, Korea) for the training. Since the model accuracy highly depends on the training data quality, so training data are collected in a grid where each point is separated by a distance of 1 m. Later, spline interpolation is done to generate the intermediate points. This process is carried out because the continuous data collection from a user may change the size of the data depending upon the user’s speed. Instead, data are collected at specified points and intermediate data are interpolated. The interpolated data are then used to extract the magnetic features and fed them into NNs for training. The training is done using Nvidia TitanX (Santa Clara, CA, USA) on an Intel i7 machine (San Jose, CA, USA) running with a 16.0 GB random access memory. It takes approximately 2 to 2.5 h to finish the training process. A total of 33,500 samples are used for training with a split of 0.75 for training and 0.30 for validation, while 21,850 samples are used for testing. The testing is performed with an S8 and LG G6 (LG-G600L) device.

The performance of the proposed approach is evaluated in terms of the localization error. The test data are collected along with the ground truth points. The localization error is determined using the following equation:

(6) $$\sqrt{{\left(x_{gt}-x_{pred}\right)}^{2}+{\left(y_{gt}-y_{pred}\right)}^{2}},$$

where $x_{gt}$ and $y_{gt}$ show the *x* and *y* coordinates for ground truth location, while $x_{pred}$ and $y_{pred}$ represent the location calculated using the proposed approach. It is noteworthy to point out that the same path has been used for training and testing experiments. However, the training data collection path and test path do not coincide with each other. The user does not strictly follow the training path and may deviate. Thus, the training data collection points on the path and testing points may be different for the experiment.

### 5.2. Results with Total Magnetic Intensity Features

This study considers a number of scenarios to evaluate the performance of the proposed approach. Traditionally, four elements of magnetic field data are utilized to perform localization: ${mag}_{x}$, ${mag}_{y}$, ${mag}_{z}$, and ${mag}_{F}$. The ${mag}_{F}$ represents the total magnetic intensity and is calculated as:

(7) $$mag_{F}=\sqrt{mag_{x}^{2}+mag_{y}^{2}+mag_{z}^{2}}.$$

**Figure 10:**
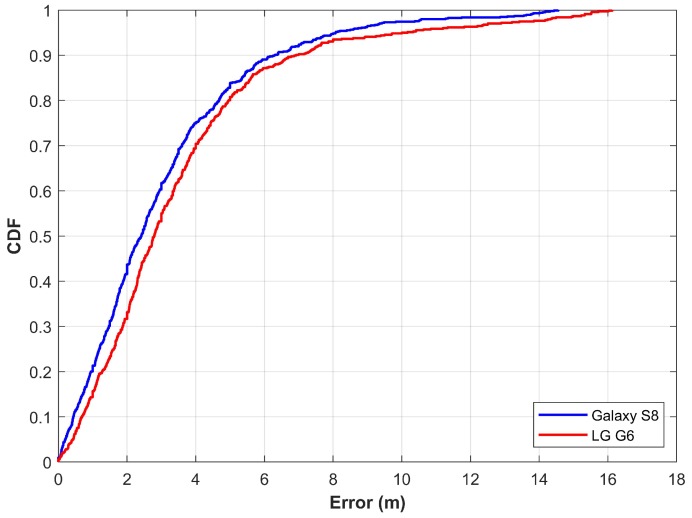
The cumulative distributive function (CDF) graph when using features from magnetic *F* alone.

The first scenario considers the experiments with $mag_{F}$ alone to extract the features for NN training. Similarly, testing is carried out in the same fashion. Results are demonstrated in Figure 10. Results demonstrate that the proposed approach is able to localize a user within 2.41 m and 2.80 m at 50% for Galaxy S8 and LG G6, respectively. Although the magnetic data samples from these smartphones are very different by magnitude, the localization results are very similar. Similarly, the localization accuracy at 75% is 4.01 m and 4.43 m for S8 and G6. Localization results are good at 50% and 75%; however, the maximum error is high, i.e., 14.55 and 16.12 for S8 and G6. Thus, this study considers the use of four elements of the magnetic field to train the NNs and predict the user’s position.

### 5.3. Results with Four Elements of Magnetic Data

**Figure 11:**
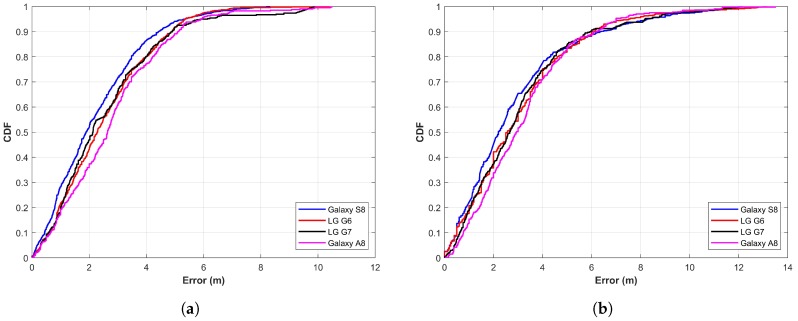
The CDF graph when using features from magnetic *x*, *y*, *z*, and *F*, (**a**) Scenario 1 and (**b**) Scenario 2.

One challenge to using the magnetic field data is that they are low-dimensional. Unlike the Wi-Fi fingerprint that has the RSS value from a number of APs at a given point, the magnetic data have only four elements to be used for fingerprints. Since the results do not meet the standards of indoor localization when only the magnetic *F* is used, in the second experiment, magnetic *x*, *y*, *z*, and *F* are considered. The features from these elements are extracted for NN training. Hypothetically, the use of a higher number of magnetic elements would yield higher localization accuracy. The authors in [31,37] also point out that the localization accuracy is higher if magnetic *x*, *y*, *z*, and *F* are used than that of magnetic *F* alone. Results are generated using four elements of the magnetic field with the proposed approach and displayed in Figure 11. Results show that the use of four magnetic elements has improved localization accuracy. The localization accuracy at 50% is now 1.89 m, 2.27 m, 2.17 m, and 2.64 m for S8, G6, G7, and Q6, respectively. In the same way, the proposed approach is able to achieve a localization accuracy of 3.75 m at 75% irrespective of the smartphone when magnetic *x*, *y*, *z*, and *F* are used. The error goes higher than 6 m only after 94.96%. Similarly, the maximum error has also been reduced to 8.32 m, 10.44 m, 9.88 m, and 10.48 m for S8, G6, G7, and Q6, respectively. Although the localization accuracy is slightly different for the four devices used for the experiment, it is very similar, which reveals that the approach is less affected by the change of smartphone. Results demonstrate that the proposed approach achieves the goal of the study, which is to devise a method that can show very similar localization results with various smartphones when the magnetic data are used for localization.

Table 2 shows the statistics for localization with features from $mag_{F}$ alone and features from four elements of magnetic data for scenarios 1 and 2. It reveals that mean error as well as the standard deviation and maximum error are high when localization is performed with $mag_{F}$ alone. The localization accuracy is better with four components of the magnetic field, as the feature vector is high-dimensional compared to that of $mag_{F}$ alone. When NNs are fed with more features, they perform better and localization accuracy is high. Scenario 2 adds three laboratories that add complexity to user walking movements along with different directions. It degrades the performance and the mean error is increased as a result. Even so, the proposed approach is able to predict the user within 4 m using two different devices at 75%.

### 5.4. Localization Results with Continuous Walk Training Data

**Figure 12:**
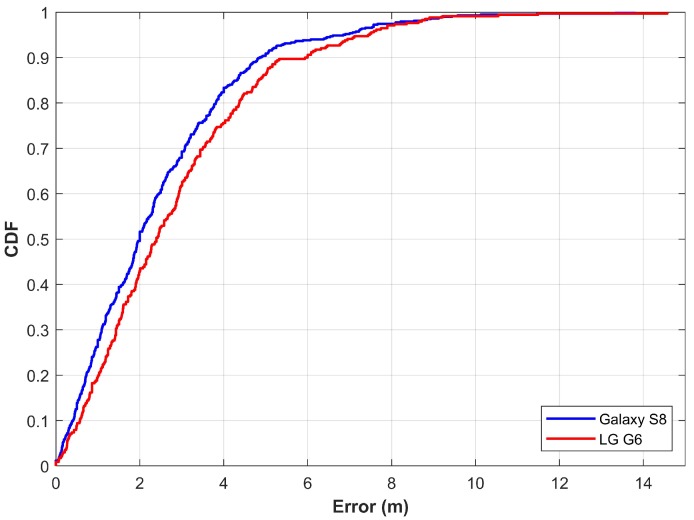
The CDF graph when localizing with features extracted from continuous walk data.

As pointed out in Section 5.1, the training data are collected at grid points and later interpolated to extract features for training. However, this study considers data collection with a continuous walk in the area of localization. For this purpose, the path shown in Figure 9a is followed with Galaxy S8 held in the hand. During the data collection, a user walks continuously along the path at a medium pace without stopping. It is important to point out that only the training data collection scenario has been changed from ‘point to point data collection’ to ‘continuous data collection’. The testing procedure remains the same. The testing process involves the localization when the user is walking on the test path. The rest of the procedure is the same for both training and testing. The purpose of this experiment is to analyze if the difference in training data collection impacts the localization accuracy. Figure 12 shows the results for the scenario where the training data are collected with a continuous walk.

Detailed statistics for the experiment are shown in Table 3. Results show that the localization performance is slightly degraded when the training data are collected with a continuous walk. Each time the data are collected, the data samples may be different depending upon the walking speed of the user. Moreover, the walking speed may vary during the walk at different locations, which affects the extracted features. It increases the localization error. Thus, the mean, as well as maximum error, are higher for this scenario. However, the proposed approach is able to localize a user withing 3.94 m at 75% with two different devices.

### 5.5. Localization Results with Publicly Available Magnetic Data

**Figure 13:**
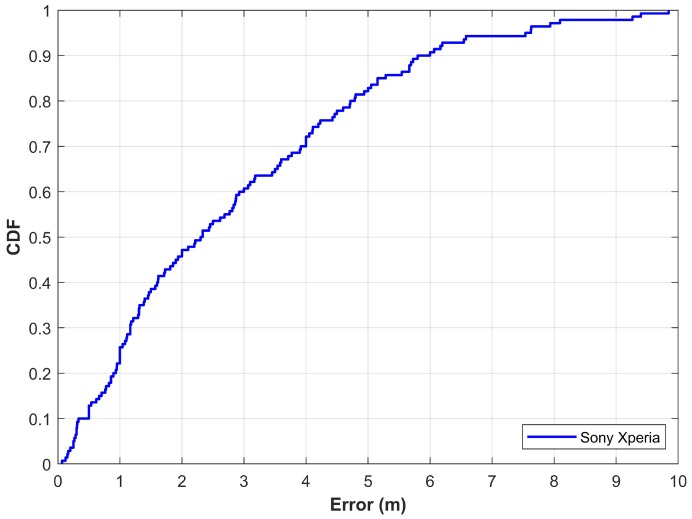
The CDF graph for localization with magnetic data from Barsocchi et al. [56].

Most of the researchers evaluate their proposed approach on their private datasets where the reported results cannot be regenerated. The current study, however, makes use of a publicly available dataset to evaluate the performance of the proposed approach. For this purpose, the magnetic dataset introduced by Barsocchi et al. in [56] has been used. The dataset was presented in an indoor positioning and indoor navigation conference held in 2016. It contains the magnetic data collected with a Sony Xperia M2 (Minato Tokyo, Japan) within a building that contains offices, corridors, and open halls. The dataset contains a total of 36,495 magnetic samples continuously collected for an indoor environment of 185.12 m^2^ at a sampling rate of 10 Hz. The current study uses 70% data for training and 30% for testing purposes. Experiment results using the proposed approach with this data are shown in Figure 13. Results demonstrate that the proposed approach shows promising localization accuracy. The mean error is 2.84 m with a standard deviation of 2.24 m, while the maximum error is 9.84 m with a Sony Xperia smartphone. The localization error is 2.33 m and 4.20 m at 50% and 75%, respectively. The performance of the proposed approach using Sony Xperia is very similar to that of using smartphones including Galaxy S8, LG G6, LG G7, and Galaxy A8. Although the smartphones used are equipped with different embedded magnetometers, the localization results are very similar, which proves that the proposed approach can potentially mitigate the impact of various devices on localization accuracy.

### 5.6. Impact of Various Device Attitudes on Localization Accuracy

**Figure 14:**
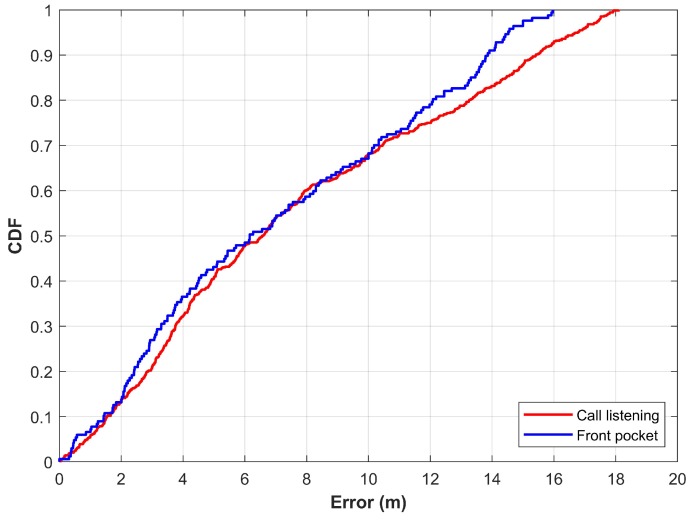
CDF graph for ’call listening’ and ’front pocket’ attitudes.

As described in Section 3, various attitudes of even the same device have a significant change in the magnetic readings. This study considers attitudes of ’call listening’, and ’front pocket’ in addition to the regular mode of walking. Figure 14 shows the results for these attitudes using the proposed approach.

The results show that the change in device attitude degrades the localization accuracy. The detailed statistics for three attitudes of the device are given in Table 4. It reveals that the mean error, in addition to the standard deviation and 50% error, is increased when localization is performed with different attitudes of the device. The important point to explain such differences is the change in the magnetic readings when the device’s attitude is changed. Since the features are extracted from the magnetic data, a change in magnetic data changes the extracted features, which, in turn, causes the erroneous predictions of user location. One possible solution to overcome this problem is to utilize separate NN for various attitudes of a device. Furthermore, the introduction of a module that could identify the device attitude can help to determine which NNs are to be used for localization.

### 5.7. Performance Analysis

The results of the proposed approach have been compared with SVM and other magnetic positioning approach called mPILOT [39]. The approach presented in [39] makes use of PDR data and magnetic field data. It works with the magnetic fingerprint database technique and a total of 14 s data to calculate the current position of the user.

**Figure 15:**
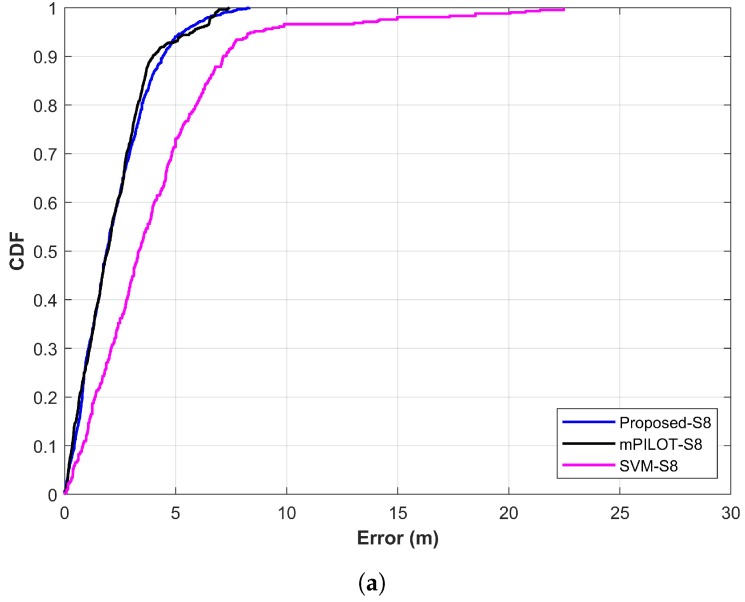

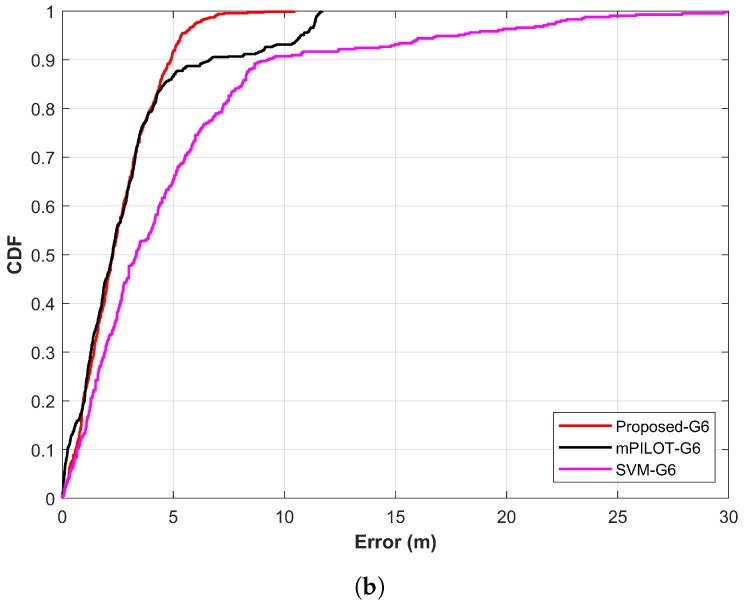
Comparison of proposed approach with mPILOT [39] and SVM: (**a**) Galaxy S8 results and (**b**) LG G6 results.

The SVM is used as well to analyze the performance of the proposed approach. Support vector machines are one of the widely used techniques for classification and regression problems proposed by Cortes and Vapnik [57,58]. It is basically designed to solve binary class problems; however, it can be used for multi-class classification too. For this purpose, it follows the “one to one” strategy [59]. This study uses it for multi-class classification and utilizes the top *k* predictions just like the NNs used in the study. The localization process is the same for NNs and SVM. Figure 15a,b shows the comparison of localization results for Galaxy S8 and LG G6 separately for the proposed technique, mPILOT, and SVM. Accuracy comparison reveals that the proposed approach outperforms mPILOT and SVM. Apparently, Figure 15 seems to show very similar localization accuracy for the proposed and mPILOT approaches; however, the important factor to decide the superiority of the approaches is the amount of data used for localization. As stated above, mPILOT uses 14 s of magnetic data. On the other hand, the proposed approach utilizes only three frames of 2 s data (6 s data in total). In other words, the proposed approach is able to achieve the same/better accuracy than that of mPILOT, with only 40% of the data used by mPILOT. The SVM shows poor performance compared to that of the proposed and mPILOT techniques. The detailed statistics for accuracy comparison are shown in Table 5.

## 6. Conclusions

This study presents the use of deep neural networks (NN) to perform magnetic field-based indoor localization using heterogeneous devices. Wi-Fi based indoor positioning systems are unable to meet the requirements of fast-paced location-based services due to intrinsic limitations and dynamic environmental factors. Magnetic field-based positioning systems (MPS) have emerged as a potential candidate for indoor positioning; however, they are limited by the use of multifarious devices which impair their wide use. Contrary to conventional MPS that makes use of the magnetic data, this study leverages the features extracted from the magnetic data. An ensemble approach is proposed where the predictions from three NN along with the pedestrian dead reckoning (PDR) are exploited to locate a user. Experiments are conducted on Galaxy S8, LG G6, LG G7, and Galaxy A8 devices, where the NN are trained on Galaxy S8 data alone. Results demonstrate that the proposed approach potentially mitigates the impact of the device change. The localization accuracy is 2.64 m at 50% and 3.75 m at 75% without regard to the localization device used. Furthermore, results with a publicly available magnetic data from Sony Xperia M2 corroborate the performance of the proposed approach and show a localization error of 2.33 m and 4.20 m at 50% and 75%, respectively. Results prove that the use of four elements of magnetic data i.e., magnetic *x*, *y*, *z*, and *F* produce high localization accuracy than that of using magnetic *F* alone.

## 7. Limitations and Future Work

Although the proposed approach shows promising results even with four different devices, yetit is not without its demerits. The experiments are carried out with a fixed device attitude that is a common assumption for the majority of works in magnetic field-based localization. The localization accuracy is degraded when the device’s attitude is changed. One possible solution to tackle this issue is to train multiple neural networks (NN) and use them accordingly. The addition of the device attitude identification module could help to determine which NN to utilize for the localization. Additionally, more devices are planned to be tested with the proposed approach.

## Figures and Tables

**Table 1:** Features extracted from magnetic data.

Features	Equation
Minimum	min=min(magnetisamples)
Maximum	max=max(magnetisamples)
Mean	$\mu =\frac{1}{n}\sum_{i=1}^{n}x_{i}$
Trimmed mean	$\mu_{0.25}=\frac{1}{n}\sum_{i=1}^{n}x_{i}$
Median	$\tilde{x}=\frac{x\lceil nx÷2\rceil +x\lceil nx÷2+1\rceil }{2}$
Root mean square	${rms}_{x}=\sqrt{\frac{1}{n}\left(x_{1}^{2}+x_{1}^{2}+\ldots +x_{n}^{2}\right)}$
Standard deviation	$\sigma =\sqrt{\frac{1}{n}\sum_{i=1}^{n}{\left(x_{i}-\mu \right)}^{2}}$
Interquartile	$IQR=\frac{3}{4}{\left(n+1\right)}^{th}-\frac{1}{2}{\left(n+1\right)}^{th}$
Percentiles (1, 50, 75, 99)	$i=\frac{{np}_{i}}{100}+0.5$
Mean absolute deviation	$MAD=\frac{\sum_{i=1}^{n}\left|x_{i}-\bar{x}\right|}{n}$
Kurtosis	$k=\left\{\frac{n(n+1)}{\left(n-1\right)\left(n-3\right)\frac{\sum_{i=1}^{n}{\left(x_{i}-\tilde{x}\right)}^{4}}{\sigma^{4}}}\right\}-\frac{3{\left(n-1\right)}^{2}}{\left(n-2)(n-3\right)}$
Coefficient of variance	$\hat{C_{v}}=\frac{\sigma }{\tilde{x}}$
Shanon’s entropy	$H\left(X\right)=-\sum_{i=0}^{N-1}p_{i}{log}_{2}p_{i}$
Skewness	${Sk}_{p}=\frac{\bar{x}-mode}{\sigma }$
Variance	$\sigma^{2}=\frac{1}{n}\sigma_{i=1}^{n}{\left(x_{i}-\mu \right)}^{2}$

**Table 2:** Results statistics for single vs. multiple features based prediction.

Features Used	Device	Mean Error	Standard Deviation	75% Error	Maximum Error
$mag_{F}$	Galaxy S8	3.02	2.61	4.00	14.55
	LG G6	3.52	3.01	4.43	16.12
	Galaxy S8	2.23	1.62	3.21	8.32
$mag_{x},mag_{y},mag_{z},mag_{F}$	LG G6	2.52	1.65	3.55	10.44
( Scenario 1)	LG G7	2.59	1.96	3.47	9.88
	Galaxy A8	2.78	1.83	3.75	10.48
	Galaxy S8	2.88	2.49	3.86	12.14
$mag_{x},mag_{y},mag_{z},mag_{F}$	LG G6	3.05	2.37	4.00	13.67
( Scenario 2)	LG G7	3.06	2.37	4.05	12.93
	Galaxy S8	3.23	2.17	4.35	13.51

**Table 3:** Results statistics when training data are collected with a continuous walk.

Device	Mean Error	Standard Deviation	75% Error	Maximum Error
Galaxy S8	2.45	2.06	3.39	13.81
LG G6	2.85	2.19	3.94	14.57

**Table 4:** Results statistics for various attitudes of the device.

Attitude	Mean Error	Standard Deviation	50% Error
Navigation	2.23	1.62	1.89
Call listening	7.59	5.10	6.54
Front pocket	7.04	4.68	6.16

**Table 5:** Results statistics for single vs. multiple features based prediction.

Device	Approach Used	Mean Error	Standard Deviation	75% Error	Maximum Error
Galaxy S8	SVM	3.34	3.41	5.24	22.47
	mPILOT	2.17	1.59	3.14	7.41
	Proposed	2.23	1.62	3.21	8.32
LG G6	SVM	4.93	5.23	6.11	29.90
	mPILOT	2.96	2.83	3.51	11.69
	Proposed	2.52	1.65	3.55	10.44
